# Patient-Reported Outcome Measures Used in Primary Hyperparathyroidism: A Scoping Review

**DOI:** 10.1245/s10434-026-19246-4

**Published:** 2026-02-12

**Authors:** Charlotte L. Leblang, Mariem Ahmed, Carrie G. Wade, Nancy L. Cho, Gerard M. Doherty, Matthew A. Nehs, Jason B. Liu

**Affiliations:** 1 Department of Surgery, Lahey Hospital & Medical Center, Burlington, MA USA; 2 Patient-Reported Outcomes, Value, and Experience Center, Mass General Brigham, Boston, MA USA; 3 Harvard Medical School, Boston, MA USA; 4 Department of Surgery, Mass General Brigham, Boston, MA USA; 5 Department of Surgical Oncology, The University of Texas MD Anderson Cancer Center, Houston, TX USA

**Keywords:** Hyperparathyroidism, Quality of life, Parathyroidectomy, Patient-reported outcome measures, Questionnaire, Survey

## Abstract

**Background:**

Standardized measuring tools, such as patient-reported outcome measures (PROMs), are needed to sufficiently measure the effect of an intervention or to describe patterns of disease. The extent to which PROMs have been used in primary hyperparathyroidism (PHPT) research is undefined. We conducted this scoping review to delineate the current landscape of PROMs used in research involving patients with PHPT.

**Methods:**

Six databases were systematically searched, and two independent reviewers identified all primary research articles that involved patients with PHPT and used PROMs. Characteristics of the identified studies and the PROMs were collated.

**Results:**

We examined 243 studies in full. Of these, 147 (49.7%) reported on symptoms or other health-related quality of life constructs that could be measured with PROMs but were not. In total, 96 (50.3%) studies used at least one PROM and were included. The median number of PROMs used per study was one (interquartile range 1–2), and 36 unique PROMs were identified. The three most frequently used PROMs were the 36-item Short Form Health Survey (42 studies), the Parathyroidectomy Assessment of Symptoms (20 studies), and the Beck Depression Inventory (12 studies). Symptoms and mental health constructs (e.g., anxiety, depression) were the most frequently assessed. Validity evidence for use in patients with PHPT was identified for only three PROMs: the 36-item Short Form Health Survey, Parathyroidectomy Assessment of Symptoms, and the Primary Hyperparathyroidism Quality of Life Questionnaire. All others were validated for use in patients without PHPT.

**Conclusions:**

There are missed opportunities in the rigor of health-related quality of life measurement in PHPT research. Future clinical research should emphasize proper application of PROMs to attain valid conclusions.

**Supplementary Information:**

The online version contains supplementary material available at 10.1245/s10434-026-19246-4.

Primary hyperparathyroidism (PHPT) is a common endocrine disease that, when left untreated, can lead to target-organ damage, including premature osteoporosis and recurrent kidney stones.^1^ Patients with PHPT can also present with symptoms such as fatigue, poor cognition, sleep disturbance, and anxiety, which negatively affect health-related quality of life (HRQL).^2–4^ The only current cure for PHPT involves surgery to remove the overactive parathyroid gland(s). In special circumstances, observation is a possible alternative. Although consensus-based guidelines outline when surgery is indicated, they do not include the potential for symptom and HRQL improvement.^5,6^

Researchers have attempted to demonstrate the effectiveness of surgery for PHPT to durably improve symptoms and HRQL, with mixed results.^2,7^ Many of these studies have relied on anecdotal clinician reports and medical record documentation. This unstructured approach makes it difficult to objectively evaluate outcomes and to compare findings. Without standardized, well‑specified outcomes and clinically relevant thresholds for interpretation, researchers and clinicians cannot determine whether observed changes are meaningful.^8^ Patient-reported outcome measures (PROMs) are standardized measuring tools that objectively quantify intangible constructs related to HRQL directly from patients without any interpretation by others.^9–12^ Although PROMs have been used to assess PHPT outcomes, the extent to which they have been used, which PROMs have been used, and whether those that have been used are appropriate for patients with PHPT remains unknown.

We conducted this review to understand the current landscape of PROMs in PHPT research. We asked two specific research questions: To what extent have PROMs been used in PHPT research, and which ones have been used? We aimed for our findings to help redirect the research conducted in this space so that progress towards rigorous health outcomes measurement can be achieved.

## Methods

This scoping review was conducted in accordance with the Joanna Briggs Institute approach and reported with the Preferred Reporting Items for Systematic Reviews and Meta-Analyses extension for Scoping Reviews (PRISMA-ScR).^13,14^ The protocol was registered on PROSPERO (CRD42023438287) and did not require ethics approval.

### Literature Search and Data Extraction

Embase, MEDLINE, CINAHL Complete, Web of Science, PsycINFO, and Cochrane Library were systematically searched from their inception to October 8, 2024, using a comprehensive search strategy formulated by a clinical librarian (CW; Supplemental Table 1). Briefly, the search strategy was designed to identify primary studies involving patients aged ≥18 years with PHPT that aimed to assess pertinent symptoms and HRQL constructs to either describe the patient cohort or as study outcomes. All PHPT types (i.e., classic, normocalcemic, normohormonal, hereditary, etc.) were included.

Search results were imported into Covidence (Melbourne, Victoria, Australia), and the titles and abstracts were independently screened by two reviewers (CL, JL) for possible inclusion. As a scoping review, primary research articles that potentially examined any HRQL construct related to PHPT that could be conceivably measured with a PROM, including any mention of “symptoms,” “symptomatic,” or “asymptomatic" were examined in full, when retrievable and in English. We also included at this stage articles with any mention of symptoms pertinent to PHPT, such as fatigue, pain, brain fog, forgetfulness, weakness, constipation, etc. for full-text review. Case reports, grey literature, protocols, editorials, commentaries, conference abstracts, reviews, consensus statements, and practice guidelines were excluded. Bibliographies of reviews, consensus statements, practice guidelines, and the included studies were reviewed for additional articles.

During full-text review, articles were included if they used at least one PROM (i.e., studies that combined neurocognitive tests, PROMs, and/or other measuring tools were included to identify the PROMs used). A PROM was defined as a questionnaire, plus the information and documentation that support its use, to measure the effect of a medical intervention on one or more constructs (e.g., a symptom or group of symptoms, effects on a particular function or group of functions, or a group of symptoms or functions shown to measure the severity of a health condition) directly from the patient with any assistance or interpretation from a healthcare professional.^10–12,15^ *Ad hoc* surveys, questionnaires, or “yes/no” symptom checklists created by study investigators were by definition excluded. PROMs that were modified in any way were excluded. PROMs that were completed with the assistance of interviewers or proxies were excluded unless the PROM was specifically validated for interviewer or proxy administration. We made certain to distinguish self-administered neurocognitive tests (e.g., Lumosity©) from PROMs using an exhaustive online search of the measuring tool if the study did not provide enough detail. Two reviewers (CL, JL) abstracted data independently. Disagreements between the two reviewers (CL, JL) were resolved through discussion or adjudication by a third reviewer (MA).

We extracted and summarized data on the publication year, country of origin, journal type, study design, sample size, proportion of female patients, PHPT types, whether a PROM was used as a primary study outcome, and study objectives and conclusions.

As a scoping review, quality assessment of studies (i.e., certainty of evidence) was not performed. Rather, the PROMs used in each study were identified and examined. Original copies of each PROM and its scoring instructions were obtained for analysis, whenever possible. For instance, proprietary PROMs and their scoring manuals were not obtained if licensing or purchasing fees were required. PROM development papers, citations from the included studies, PROQOLID™,^16^ and the COSMIN Database of Systematic Reviews were searched exhaustively to extract data and ensure included PROMs were legitimate.^17^ Characteristics of the PROMs were tabulated and summarized qualitatively or quantitatively, where appropriate, following data synthesis methods as outlined by the Joanna Briggs Institute.^13^ We extracted the following data on identified PROMs: name, development year, country of origin, target population, category or PROM type,^9^ constructs measured, number of items, psychometric theory (classical test theory [CTT], Rasch measurement theory, item response theory [IRT]), and whether validity evidence for use in patients with PHPT was available. We determined whether any validity evidence existed by identifying any available studies reporting on the measurement properties of PROMs for patients with PHPT.

## Results

**Fig. 1:**
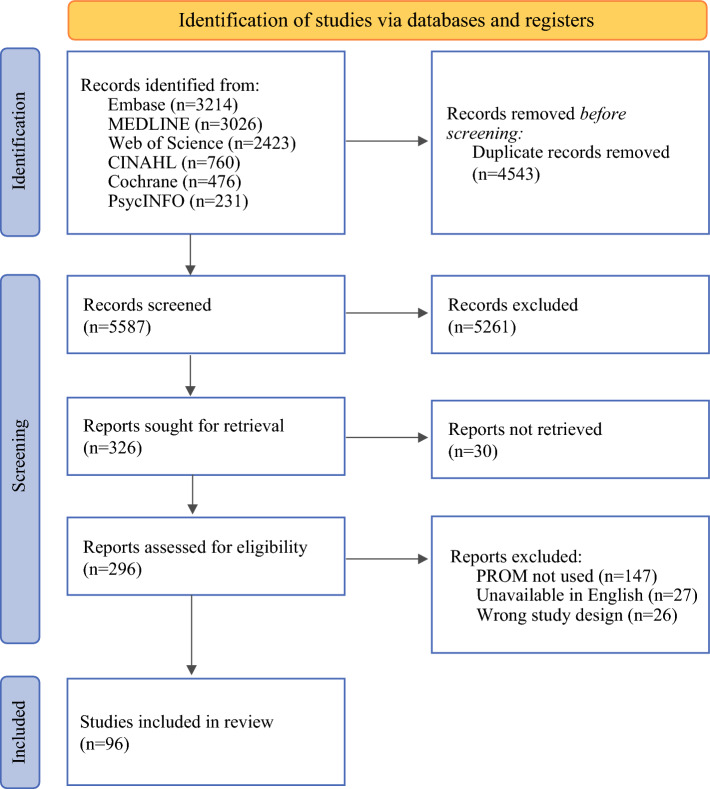
Preferred Reporting Items for Systematic Reviews and Meta-Analyses (PRISMA) diagram. PROM, patient-reported outcome measure

After removing duplicates and screening for inclusion, 326 articles were sought for full-text retrieval (Fig. 1). In total, 30 could not be located or obtained, 27 were not in English, and 26 did not meet inclusion based on the study design criterion, yielding 243 studies that were examined in full. Of these, 147 (49.7%) reported on symptoms or other HRQL constructs that could be measured with PROMs but were not. Six (4.1%) used neurocognitive tests only and 141 (95.9%) tracked symptoms using ad hoc questionnaires, checklists, or medical record documentation. A total of 96 (50.3%) studies used at least one PROM and were included.

### Study Characteristics

**Fig. 2:**
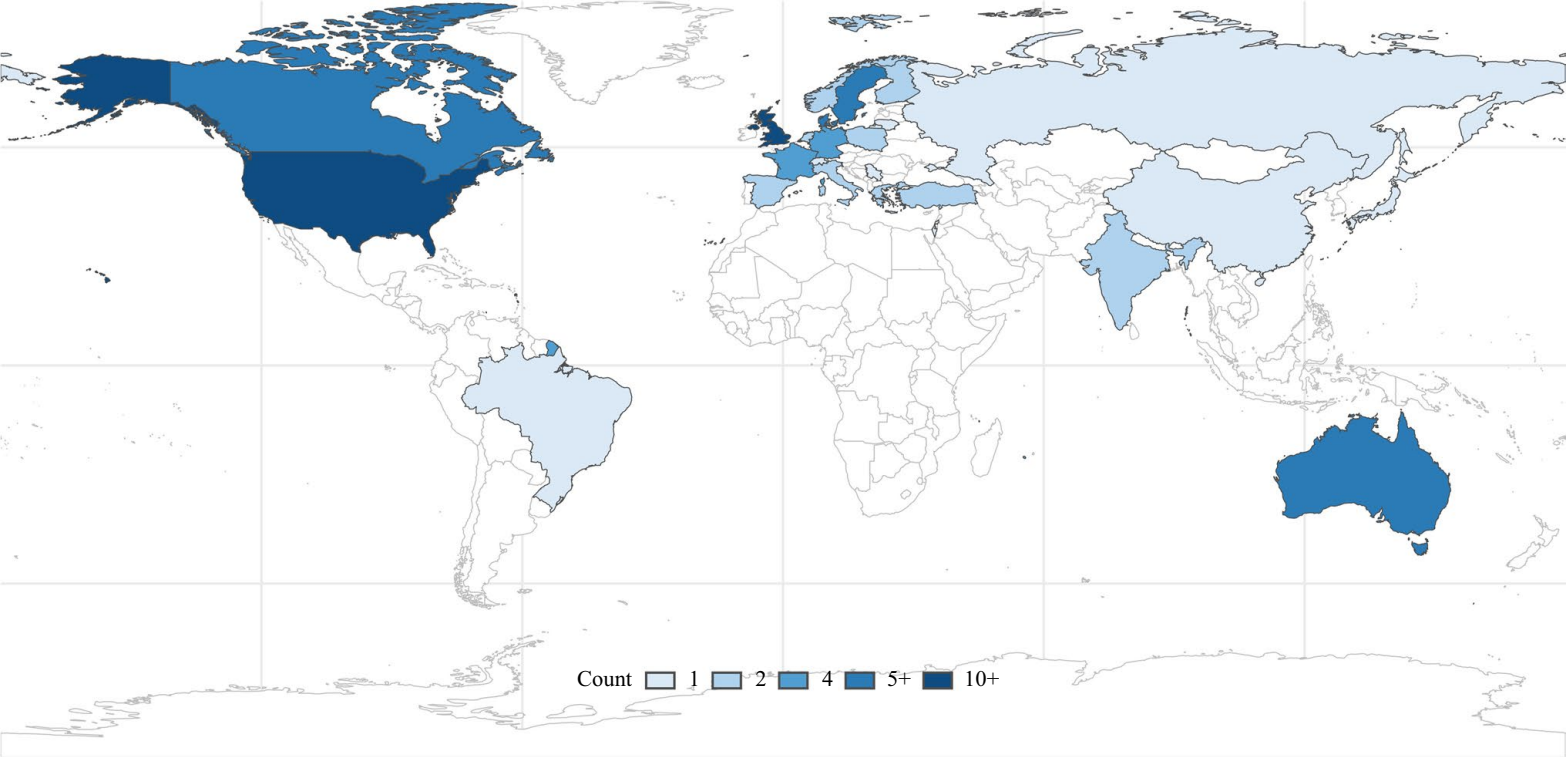
Number and distribution of included studies (*n*=96) across the globe

Table 1 summarizes the study characteristics, and all included studies are reported in Supplemental Table 2. The earliest published study that used a PROM was in 1991.^18^ Studies that used PROMs were most frequently published in general surgical journals (52%). Prospective cohort study designs were predominant (59%). Most studies included patient cohorts from the USA (37 studies; Figure 2), followed by the UK (10 studies). Patients with classic/hypercalcemic PHPT were the most common (82 studies). Across studies, the median sample size was 72 (interquartile range [IQR] 45–129), and the median proportion of female patients was 79% (IQR 74–83).

**Table 1.** Characteristics of the included studies

Characteristic	Counts
*Publication year*	
1989 or earlier	0
1990–1994	2
1995–1999	3
2000–2004	13
2005–2009	22
2010–2014	18
2015–2019	16
2020–2024	22
*Study design*	
Randomized controlled trial	10
Prospective cohort	57
Case-control	21
Cross-sectional	5
Retrospective cohort	3
*Journal type*	
General medical	3
Medical specialty	35
General surgical	50
Surgical specialty	8
Sample size	72 (45–129)
Percentage of female patients^a^	79 (74–83)
*Primary hyperparathyroidism type*	
Hypercalcemic	82
Normocalcemic	24
Normohormonal	49
Cannot be determined	14
Number of PROMs used per study	1 (1–2)
PROM used as primary study outcome	84 (88)

Data are presented as *n*, *n*(%), or median (interquartile range) unless otherwise indicated. Studies could be counted multiple times per pertinent category, so figures do not sum to 96 in all cases

^a^Five studies did not report patient sex

PROM, patient-reported outcome measure

**Table 2:** Characteristics of the identified patient-reported outcome measures (PROMs)

PROM name (short name)	Year developed; country of origin	Target population	Category	Construct(s) measured^a^ (number of items)	Psychometric theory	No. of studies that used the PROM	Published validity evidence for use in PHPT
15 Dimension health-related quality of life (15D HR-QOL)^40^	1989; Finland	General	Preference-based, universal, global	Utility index (15)	CTT	2	No
Beck depression inventory (BDI), BDI version 2 (BDI-II)^41^	1961, 1996; USA	Pts with depression	Universal, domain-specific	Depression (21)	CTT	12	No
Brief symptom inventory, 18-item^42^	2001; USA	General	Universal, domain-specific	Somatization (6), depression (6), anxiety (6), overall psychological distress (18)	CTT	1	No
Center for Epidemiologic Studies Depression Scale (CES-D)^43^	1977; USA	General	Universal, domain-specific	Depression (20)	CTT	1	No
Depression anxiety stress scale, 21-item (DASS-21)^44^	1995; Australia	General	Universal, domain-specific	Stress (7), anxiety (7), depression (7)	CTT	2	No
Eight State Questionnaire (8SQ)^45^	1976; USA	General	Universal, domain-specific	Anxiety (12), stress (12), depression (12), regression (12), fatigue (12), guilt (12), extraversion (12), arousal (12)	CTT	1	No
EORTC Quality of life questionnaire – core questionnaire (QLQ-C30)^46^	1993; multi-country	Pts with cancer	Condition-specific, global	Global health status (2), physical functioning (5), role functioning (2), emotional functioning (4), cognitive functioning (2), social functioning (2), fatigue (3), nausea/vomiting (2), pain (2), dyspnea (1), insomnia (1), appetite loss (1), constipation (1), diarrhea (1), financial difficulties (1)	CTT	1	No
Epworth sleepiness Scale (ESS)^47^	1990; Australia	General	Universal, domain-specific	“Daytime sleepiness” (8)	CTT	2	No
EQ-3L, EQ-5D, visual analog scale (EQ-VAS)^48^	1990; multi-country	General	Preference-based, universal, global	Mobility (1), self-care (1), usual activities (1), pain/discomfort (1), anxiety/depression (1), utility index (5)	CTT	3	No
Fatigue Severity Scale (FSS)^49^	1989; USA	MS, SLE, Parkinsonism, fibromyalgia	Condition-specific, domain-specific	Fatigue (9)	CTT	1	No
Female sexual function Index (FSFI)^50^	2000; USA	General	Universal, domain-specific	Desire (2), arousal (4), lubrication (4), orgasm (3), satisfaction (3), pain (3), overall (19)	CTT	1	No
Frequency scale for symptoms of gastroesophageal reflux disease (FSSG) Questionnaire^51^	2004; Japan	Pts with GERD	Condition-specific, domain-specific	Symptoms of GERD (12)	N/A	1	No
Functional outcomes of sleep questionnaire (FOSQ)^52^	1997; USA	Pts with disorders of excessive sleepiness	Condition-specific, domain-specific	Activity level (9), vigilance (7), intimacy (4), general productivity (8), social outcome (2), overall sleepiness of daily activities (30)	CTT	1	No
GERD health-related quality of life (GERD-HRQL) questionnaire^53^	1996; USA	Pts with GERD	Condition-specific, domain-specific	Symptom severity (9)	CTT	1	No
General anxiety disorder, 7 item (GAD-7) scale^54^	2006; USA	General	Universal, domain-specific	Anxiety (7)	CTT	1	No
General health questionnaire, 28-item (Ghq-28)^55^	1979; UK	Pts with anxiety disorders and depression	Condition-specific, domain-specific	Somatic symptoms (7), anxiety and insomnia (7), social dysfunction (7), depression (7)	CTT	1	No
Glasgow benefit inventory (GBI)^56^	1996; UK	Pts who underwent middle ear surgery, cochlear implant, mastoid procedures, rhinoplasty, and tonsillectomy	Condition-specific, domain-specific	General (12), social (3), physical (3), overall (18)	CTT	1	No
Health outcomes institute health status questionnaire 2.0 (HSQ-39)^57^	1996; USA	General	Universal, domain-specific	Health perception (5), physical functioning (10), role-physical (4), bodily pain (2), energy/fatigue (4), social functioning (2), role-mental (3), mental health (5), health transition (1)	CTT	2	No
Hopkins symptom checklist, 58-item (HSCL-56)^58^	1974; USA	Psychiatric pts	Condition-specific, domain-specific	Somatization (12), obsessive-compulsive (8), interpersonal sensitivity (7), anxiety (6), depression (11)	CTT	1	No
Hospital anxiety and depression scale (HADS)^59^	1983; USA	Hospitalized pts	Condition-specific, domain-specific	Depression (7), anxiety (7), overall (14)	CTT	7	No
Insomnia severity index (ISI)^60^	1985; Canada	Pts with insomnia	Condition-specific, domain-specific	Overall symptom severity (7)	CTT	3	No
Kellner symptom questionnaire (KSQ)^61^	1976; USA	General	Universal, domain-specific	Depression overall (23), anxiety overall (23), hostility overall (23), somatization overall (23), depression-symptom (17), anxiety-symptom (17), hostility-symptom (17), somatization-symptom (17), contentment (6), relaxation (6), friendliness (6), physical well-being (6)	N/A	1	No
Medical outcomes study/RAND 36-item short form health survey (Sf-36)^62^	1990, 1998; USA	General	Universal, global, domain-specific	Physical functioning (10), social functioning (2), role-physical (4), role-emotional (3), mental health (5), vitality (4), pain (2), general health (5), health transition (1) physical health component (21), mental health component (14)	CTT	42	Yes
Medical outcomes study/RAND 12-item short form health Survey (SF-12)^63^	1995, 2000; USA	General	Universal, global	Physical health component (6), mental health component (6)	CTT	1	No
Parathyroidectomy assessment of symptoms (PAS)^64^	1998; Canada	Pts with PHPT	Condition-specific, domain-specific	Symptom presence and severity (13)	CTT	20	Yes
Patient health questionnaire, 9-item (PHQ-9)^65^	2001; USA	General	Universal, domain-specific	Depression (9)	CTT	5	No
Patient-reported outcomes measurement information system (PROMIS)^66^	2005; USA	General	Universal, condition-specific, domain-specific, global	Physical health (12 constructs), mental health (15 constructs), social health (7 constructs), global health (2 constructs) (see healthmeasures.net)	IRT	2	No
Pittsburgh sleep quality index (PSQI)^67^	1989; USA	General	Universal, domain-specific	Overall sleep quality (19)	CTT	4	No
Positive state of mind (PSOM) scale^68^	1988; USA	General	Universal, global	Focused attention (1), productivity (1), responsible caretaking (1), restful repose (1), sensuous nonsexual pleasure (1), sharing (1), sensuous sexual pleasure (1), overall (7)	CTT	1	No
Primary hyperparathyroidism quality of life (PHPQoL) Questionnaire^69^	2013; Spain	Pts with PHPT	Condition-specific, domain-specific	Physical functioning (9), emotional functioning (7)	Rasch	4	Yes
Sickness impact profile, 68-item (SIP-68)^70^	1994; the Netherlands	General	Universal, domain-specific	Somatic autonomy (17), mobility control (12), psychological autonomy and communication (11), social behavior (12), emotional stability (6), mobility range (10)	CTT	1	No
State-trait anxiety inventory-AD (Form Y) (STAI-AD)^71^	1983; USA	General	Universal, domain-specific	Anxiety state (20), anxiety trait (20)	CTT	6	No
Swallowing quality of life, 44-item (SWAL-QOL)^72^	2002; USA	Pts with dysphagia	Condition-specific, domain-specific	Burden (2), eating duration (2), eating desire (3), symptom frequency (14), food selection (2), communication (2), fear (4), mental health (5), social (5), fatigue (3), sleep (2)	CTT	2	No
Symptom checklist-90-revised (SCL-90-R)^73^	1975; USA	Psychiatric Pts	Condition-specific, domain-specific, global	Somatization (12), obsessive-compulsive (10), interpersonal sensitivity (9), depression (13), anxiety (10), hostility (6), phobic anxiety (7), paranoid ideation (6), psychoticism (10), global severity index (1), positive symptom total (1), positive symptom distress index (1), overall (84)	CTT	4	No
Voice handicap index, 10-item (VHI-10)^74^	2004; USA	Pts with voice disorders	Condition-specific, global	Voice handicap (10)	CTT	2	No
World health organization-five well-being index (WHO-5)^75^	1998; Denmark	General	Universal, domain-specific	Well-being (5)	CTT	3	No

^a^Constructs were determined based upon measure scoring instructions

CTT, classical test theory; EORTC, European Organisation for Research and Treatment of Cancer; GERD, gastroesophageal reflux disease; IRT, item response theory; MS, multiple sclerosis; N/A, not applicable; PHPT, primary hyperparathyroidism; pts, patients; SLE, systemic lupus erythematosus

### PROMs

Of the 96 studies, the median number of PROMs used per study was one (IQR 1–2). A PROM was used as the primary outcome in 84 (88%) studies, and the remaining studies used a PROM as a secondary outcome.

**Fig. 3:**
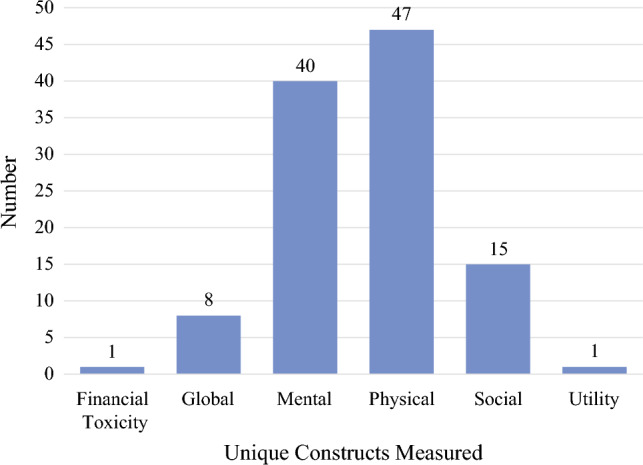
Summary of constructs measured across the 36 identified patient-reported outcome measures

In total, 36 unique PROMs were identified (Table 2). A potential PROM called the “Brief Sleep Disturbance Inventory” reported in one study could not be identified.^19^ The three most frequently used PROMs were the 36-item Short Form Health Survey (SF-36; 42 studies), the Parathyroidectomy Assessment of Symptoms (PAS; 20 studies), and the Beck Depression Inventory (12 studies). Figure 3 summarizes the 112 unique constructs across the 36 PROMs identified into four overarching categories; specific constructs are listed in Table 2. Symptoms and mental health constructs (e.g., anxiety, depression) were the most frequently assessed.

Validity evidence for use in patients with PHPT was identified for only three (8.3%) PROMs: the SF-36, PAS, and the Primary Hyperparathyroidism Quality of Life Questionnaire (PHPQoL).^20^ All other PROMs were validated for use in other non-PHPT patient populations.

Two PROMs were psychometrically developed using modern measurement theory: the Patient-Reported Outcomes Measurement Information System (PROMIS®) and the PHPQoL. These were used in two and four studies, respectively. Validation evidence could not be identified for two PROMs: the Frequency Scale for Symptoms of Gastroesophageal Reflux Disease Questionnaire and the Kellner Symptom Questionnaire. Two PROMs intended to be used as health utility indices (i.e., preference-based measures) were used as global HRQL measures: the 15 Dimension Health-Related Quality of Life tool and the EQ-5D.^21^

## Discussion

PHPT is a chronic neoplastic condition that can cause, for some, a constellation of symptoms negatively affecting HRQL. Symptoms and HQRL can be objectively and reproducibly measured using PROMs, but the extent to which PROMs have been used in PHPT research has not been comprehensively characterized. This scoping review identified 36 unique PROMs from 96 studies. Only three PROMs were specifically developed or validated for use in this population. Nearly half of the studies that we identified reported symptoms or made claims about HRQL without using PROMs, representing gaps in scientific rigor and missed opportunities. These deficiencies contribute to the ongoing dispute as to whether surgery for PHPT can improve symptoms and HRQL.

Patient-reported outcomes (PROs) are defined as any reports coming directly from patients about how they function or feel in relation to a health condition and its therapy, without interpretation of the patient’s responses by a clinician or anyone else.^10–12^ PROs can include symptoms, perceived health, and overall HRQL. A PROM is the measuring tool or instrument that quantifies PROs into numerical data that can be interpreted like any other clinical data, such as a laboratory test. Using the search strategy in this scoping review, approximately half of all studies quantified symptoms or other aspects of HRQL without using any PROMs, underscoring substantial inconsistency in methodological rigor across the literature. In these studies, symptoms or HRQL were commonly reported using unstructured or non-standardized methods, such as retrospective medical record or documentation reviews, clinician assessments, or symptom tallies. This lack of standardized assessment introduces measurement heterogeneity that limits direct comparison of data across studies, complicates evidence synthesis, and weakens the overall quality of clinical evidence available. Furthermore, the inconsistent use of PROMs may delay the identification of clinically meaningful treatment effects and obscure accurate assessments of symptom burden, potentially contributing to the widespread under-recognition and undertreatment of PHPT.^4,22–24^ For example, the use of “yes/no” symptom checklists inherently necessitates complete symptom resolution to determine “success” when an improvement without resolution could be meaningful to patients. Collectively, these gaps suggest an underappreciation of the importance of standardized psychometric assessments in PHPT research and highlight the need for greater methodological rigor, clarity, and consensus around the use of PROMs in future studies.

Our review also identified a notable deficiency in PROMs rigorously validated for use in PHPT. This aligns with findings from a recent systematic review by Liang et al.,^20^ which highlighted that the three PROMs with any validity evidence (i.e., SF-36, PAS, and PHPQoL) actually lack robust psychometric properties, including deficiencies in validity, reliability, and responsiveness. In other words, although we identified three PROMs validated for use in patients with PHPT, according to Liang et al.,^20^ those PROMs need to be further validated with additional evidence. Without high certainty evidence that the PROM measures what it is intended to measure in the intended population, it may lack sensitivity to change and risk yielding misleading conclusions about treatment effectiveness. The absence of disease-specific PROMs with high-certainty validity evidence also reduces their credibility in regulatory and policy contexts, where psychometric robustness is essential for inclusion in performance measurement or labeling claims.^9,11,12^ To address this gap, future PHPT research should prioritize the development and validation of PROMs using established frameworks such as COSMIN, ensuring that instruments meet criteria for validity, reliably, and responsiveness.^17,25^

Another finding from our review was the use of global measures. These measures, which aim to capture “overall health” or “overall HRQL,” are identical to other composite measures in healthcare (e.g., the Patient Safety Indicator 90^26^). Such instruments may fail to capture symptoms and HRQL constructs relevant to patients with PHPT, leading to misclassification of symptom severity and blunting the ability to detect clinically meaningful changes.^21,27^ This limitation can compromise both the interpretability of clinical research outcomes and the accuracy of patient-centered assessments in routine care.^15^ Deliberate, methodologically sound PROM selection is critical to advancing patient-centered care and accurately quantifying outcomes that matter to patients with PHPT.^9^

Among the 36 PROMs identified, only two were developed using modern measurement methods, such as IRT or Rasch measurement theory; the remainder relied exclusively on CTT, representing older legacy measures.^28–31^ CTT, though prevalent and familiar, assumes that each score reflects a “true” score plus random error, so reliability and validity depend on the specific sample and set of items used, limiting generalizability and precision across different populations or clinical contexts.^31^ In contrast, modern psychometric approaches, such as IRT and Rasch analysis, model the probability of a response as a function of both a patient’s underlying trait level (e.g., symptom severity) and each item’s properties (e.g., difficulty). This approach places scores on a common, interval-level scale that is independent of the specific sample or item set, allowing for shorter yet more precise assessments.^29,30,32^ Despite these methodological advances, our findings demonstrate a continued reliance on older PROMs in PHPT research, often selected due to historical precedence, familiarity, convenience, or widespread prior use rather than careful consideration of their psychometric appropriateness or sensitivity. That is, CTT-based PROMs may fail to capture the subtle but clinically meaningful symptom changes after parathyroidectomy, leading researchers to conclude that there is no effect when in fact the wrong measuring tool was used. Such "off-the-shelf" selection of outcome measures can inadvertently lead to measurement imprecision, reduced sensitivity to clinically meaningful changes, and potential inaccuracies in evaluating symptom severity, treatment effects, and HRQL outcomes.^31,33^ Just as surgery requires choosing the most appropriate state-of-the-art surgical instrument for the specific scenario, rigorous measurement in research similarly demands careful consideration and critical selection of PROMs fit for their intended purpose. Future PHPT research would substantially benefit from deliberate selection of appropriate PROMs suited for purpose.

There are limitations to this study. We could not identify the full texts for 30 (9%) articles despite access to resources from three independent medical libraries. Thus, additional PROMs may have been missed. We also only examined full-text articles that were available in English, which was the only language that members of the research team could comprehend. Third, we could not identify one potential PROM related to sleep disturbance despite an exhaustive search. This may reflect that the PROM used in the research study was not a legitimate one. Fourth, publication bias and outcome reporting bias are possible, leading our results to be underestimates. Fifth, we were unable to make valid claims about PROM score changes, biochemical changes, or other specific study conclusions because of the heterogeneity of studies included. Lastly, it was not possible to determine why nearly half of all studies included in this review did not use PROMs.

Another important observation from this review was that most studies had small sample sizes. Although we did not set out to assess statistical rigor, it is important to recognize that, in any research study, sample size affects the ability to detect differences between groups, if there is one. In PROMs-based research, use of the minimally important difference, minimal clinically important difference, or other anchor-based effect sizes may be more pertinent to interpret results than frequentist statistical tests of significance.^34^ Furthermore, small sizes can limit the generalizability of research findings. In PROMs-based research, which inherently requires patients to respond, the response rate is often held as gospel. However, it is a fallacy to assume that a high response rate alone is sufficient, as the intent of the response rate is to ensure sufficient representativeness for generalizability.^35,36^ Future PHPT research involving PROMs should also consider addressing nonresponse bias.

To advance the research conducted in this space, opportunities for improvement include (a) consistently using PROMs when assessing symptoms or HRQL in PHPT research; (b) defining important PHPT-specific domains with patients and clinicians; (c) leveraging IRT-based measures to ensure interval scaling, appropriate measurement of subtle health aspects, and cross-group invariance; and (d) deploying short forms or computerized adaptive testing to reduce patient burden while increasing measurement precision.^37^ Using PROMs, especially IRT-based ones, in PHPT research would sharpen surgical decision-making (e.g., identifying which patients will most likely benefit), improve postoperative monitoring (e.g., detecting small but meaningful gains after parathyroidectomy), and enable standardized outcome comparisons across surgeons and centers (e.g., development of core outcome sets).

When integrated into routine practice, PROMs can improve patient care quality, likely through the additional two-way communication PROMs can add, such as symptom reporting.^38^ This was more commonly reported in patients who underwent routine and consistent measurements with the PROMs. Even simple reporting of symptoms via PROMs can improve patient satisfaction and communication of their symptoms to their provider.^39^ HRQL can be impacted by the symptomology the disease can elicit, and in PHPT it is possible that the use of PROMs in routine care could positively affect patients’ HRQL as they progress through treatment. The use of PROMs in PHPT may improve patient satisfaction in their care, as has been shown in other diseases with a heavier symptom burden. An emphasis on the specialization of PROMs for individual diseases may have a large impact on patient-centered care and satisfaction.

In this scoping review, nearly half of the studies purporting to measure PROs, including symptoms and HRQL, in patients with PHPT did not use PROMs as standardized measuring tools. There is little consistency across studies in the constructs measured and the types of PROMs used. This heterogeneity means that any effort to standardize or compare outcomes is challenging. Additionally, we identified only three PROMs designed for or validated for use in patients with PHPT. Further work is needed to better understand how to best measure the subtle symptoms and HRQL concerns of patients with PHPT and to develop the correct tools for their assessment.

## Supplementary Information

Below is the link to the electronic supplementary material.

Supplementary file1 (PDF 470 KB)

Supplementary file2 (PDF 302 KB)
