# Supplementary material for: Patient-Reported Outcome Measures Used in Primary Hyperparathyroidism: A Scoping Review
Source: Ann Surg Oncol. 2026 Feb 12;33(4):3435–48. doi: 10.1245/s10434-026-19246-4 (PMC12982322; doi:10.1245/s10434-026-19246-4)
Supplement: Supplementary file 1 — Supplementary file1 (PDF 470 KB) [file 10434_2026_19246_MOESM1_ESM.pdf]

Supplemental Table 1. Comprehensive search strategy.
Searches run on 2024-10-08
No search limits applied

DATABASE (PLATFORM)	SEARCH
**MEDLINE (OVID)**	1. (Parathyroidectomy/ OR exp Parathyroid Glands/su OR exp Hyperparathyroidism/su) OR (surgery OR parathyroidectom*).ti,ab,kf,kw. OR (surgical*).ti<br>2. exp Hyperparathyroidism/dt OR exp Diphosphonates/ OR Denosumab/ OR Cinacalcet/) OR (Denosumab OR biphosphonate* OR cinacalcet).ti,ab,kf,kw.<br>3. or/1-2<br>4. ("Hyperparathyroidism, Primary"/) OR ("primary hyperparathyroid*" OR pHPT OR "primary hyper-parathyroid*").ti,ab,kf,kw.<br>5. exp "Aptitude Tests"/ OR "Psychometrics"/ OR exp "Patient Health Questionnaire"/ OR exp "Neuropsychological tests"/ OR (((cognitiv* OR cognition OR intelligence OR neurocognit* OR memory OR neuropsych* OR psycholog*) adj3 (test* OR self-report* OR questionnaire* OR survey* OR answer* OR self-assess*)) OR ((patient OR patients) adj3 (assess* OR report*) adj3 (self OR outcome)) OR psychometric* OR Stanford-binet OR wechsler OR PHQ).ti,ab,kf,kw.<br>6. ("Mental Health"/ OR exp "Mental Disorders"/ OR "Neuropsychiatry"/ OR "Neuropsychology"/ OR exp "Social Isolation"/ OR "Social Inclusion"/ OR "Social Adjustment"/ OR exp "Self-Control"/ OR "Aggression"/ OR "Quality of Life"/ OR exp "Signs and Symptoms, Digestive"/ OR Fatigue/ OR Polyuria/ OR exp "Abdominal Pain"/ OR exp "Back Pain"/ OR "Neck Pain"/ OR exp Neuralgia/ OR exp Hypertension/ OR Polydipsia/ OR exp Headache/ OR exp Arthralgia/ OR exp "Musculoskeletal Pain"/)<br>7. (((drug? OR substance?) adj3 (use? OR abuse? OR misuse? OR problem? OR addict*)) OR (mental adj3 (illness OR disorder*)) OR ((sex OR sexual) adj3 (perform* OR interest* OR discomfort* OR function* OR impair* OR drive)) OR ((emotional* OR social* OR mental* OR physical*) adj3 (health OR healthy OR wellness OR wellbeing OR well-being OR support OR safety OR isolat* OR engage*)) OR (social* adj3 engage*) OR (daily adj2 activit*) OR (medication adj2 (complian* OR adheren*)) OR fatigue* OR exhaustion OR sleep* OR insomnia OR hypersomnia OR libido OR anxiety OR anxious OR depression OR depressed OR depressive OR somatoform OR "body dysmorph*" OR alcohol* OR drinking OR anger OR angry OR cognition OR cognitive* OR "mental acuity" OR concentration OR memory OR "positive affect" OR "positive outlook" OR mood OR irritab* OR frustration OR "interpersonal sensitivity" OR envy OR disagreeable OR temper OR fear OR fearful* OR panic OR misery OR worry OR dread OR hyperarousal OR tension OR nervous* OR somatic OR dizzy OR dizziness OR sadness OR sad OR guilt* OR "self esteem" OR "self worth" OR lonely OR loneliness OR "positive engagement" OR attitude OR benefit-finding OR meaning-making OR joy OR happy OR happiness OR excite* OR enthusiasm OR contentment OR psychosocial OR psycho-social OR self-efficacy OR smoking OR smoker OR cigarette* OR vaping OR vape OR e-cig OR ecig OR e-cigarette* OR ecigarette* OR tobacco OR

	marijuana OR cannabis OR THC OR nicotine OR opiate* OR heroin OR social OR companionship OR isolat* OR support* OR helpless* OR disappoint* OR symptom* OR sensorimotor OR friend* OR family OR families OR active OR activity OR sedentar* OR relationship* OR neuropsych* OR psycho* OR psychiatr* OR neurotic* OR neurocognit* OR QoL OR HRQoL OR nausea* OR vomit* OR emesis OR hyperemesis OR constipat* OR diarrhea OR thirst* OR itch OR itchy OR itching OR urination OR headache OR migraine* OR "brain fog" OR pancreatitis OR (ulcer adj3 (peptic OR stomach)) OR GERD OR gastroesophageal OR gastro-esophageal OR ((acid OR gastric) adj3 reflux) OR osteopenia* OR osteoporos* OR polyuria OR nocturia OR nephrocalcinosis OR mephorlithaiasis OR CKD OR "chronic kidney" OR hypertens* OR HTN OR "high blood pressure" OR fibromyalgia OR pruritis OR neuralgi* OR ((muscle OR muscular OR musculoskeletal OR joint OR back OR neck OR stomach OR abdomen OR abdominal OR head) adj3 (pain OR painful OR ache OR aching)) OR (quality adj3 life)).ti,ab,kf,kw.<br>8. or/5-7<br>9. 3 and 4 and 8
**EMBASE (ELSEVIER)**	1. 'parathyroidectomy'/exp OR (('hyperparathyroidism'/exp OR 'parathyroid gland'/exp) AND 'surgery'/mj) OR (surgery OR surgical* OR parathyroidectom* OR para-thyroidectom*):ti,ab,kw<br>2. ('hyperparathyroidism'/exp AND 'drug therapy'/exp/mj) OR 'cinacalcet'/exp OR 'denosumab'/exp OR 'bisphosphonic acid derivative'/exp/mj OR (denosumab OR biphosphonates OR cinacalcet):ti,ab,kw<br>3. #1 OR #2<br>4. 'primary hyperparathyroidism'/exp OR ('primary hyperparathyroid*' OR pHPT OR 'primary hyper-parathyroid'):ti,ab,kw<br>5. ('aptitude test'/mj OR 'mental test'/mj OR 'neuropsychological assessment'/exp/mj OR 'psychometry'/exp/mj OR 'cognitive impairment assessment'/exp/mj OR 'general mental disease assessment'/exp/mj OR 'mood disorder assessment'/exp/mj OR 'neuropsychiatric inventory'/exp) OR (((cognitiv* OR cognition OR intelligence OR neurocognit* OR memory OR neuropsych* OR psycholog*) NEAR/3 (test* OR self-report* OR questionnaire* OR survey* OR answer* OR self-assess*)) OR psychometric* OR stanford-binnet OR wechsler OR PHQ):ti,ab,kw OR ((patient OR patients) NEAR/3 (assess* OR report*) NEAR/3 (self OR outcome*)):ti,ab,kw<br>6. ('mental health'/exp OR 'mental disease'/exp/mj OR 'neuropsychology'/expj OR 'neuropsychiatry'/exp OR 'social connectedness'/exp/mj OR 'social isolation'/exp/mj OR 'self concept'/exp/mj OR 'aggression'/exp/mj OR 'quality of life'/exp OR 'gastrointestinal symptom'/exp/mj OR 'lethargy'/exp/mj OR 'fatigue'/exp/mj OR 'intestine function disorder'/exp/mj OR 'polyuria'/exp/mj OR 'nausea and vomiting'/exp/mj OR 'abdominal pain'/exp/mj OR 'chronic pain'/exp/mj OR 'headache and facial pain'/exp/mj OR 'limb pain'/exp/mj OR 'musculoskeletal pain'/exp/mj OR 'neuralgia'/exp/mj OR 'hypertension'/exp/mj OR 'polydipsia'/exp/mj OR 'arthralgia'/exp/mj)

	7. (((drug$ OR substance$) NEAR/3 (use$ OR abuse$ OR misuse$ OR problem* OR addict*)) OR (mental NEAR/3 (illness OR disorder*)) OR ((sex OR sexual) NEAR/3 (perform* OR interest* OR discomfort* OR function* OR impair* OR drive)) OR ((emotional* OR social* OR mental* OR physical*) NEAR/3 (health OR healthy OR wellness OR wellbeing OR well-being OR support OR safety OR isolat* OR engage*)) OR (social* NEAR/3 engage*) OR (daily NEAR/2 activit*) OR (medication NEAR/2 (complian* OR adheren*)) OR fatigue* OR exhaustion OR sleep* OR insomnia OR hypersomnia OR libido OR anxiety OR anxious OR depression OR depressed OR depressive OR somatoform OR alcohol* OR drinking OR anger OR angry OR cognition OR cognitive* OR 'mental acuity' OR concentration OR memory OR 'positive affect' OR 'positive outlook' OR mood OR irritab* OR frustration OR 'interpersonal sensitivity' OR envy OR disagreeable OR temper OR fear OR fearful* OR panic OR misery OR worry OR dread OR hyperarousal OR tension OR nervous* OR somatic OR dizzy OR dizziness OR sadness OR sad OR guilt* OR 'self esteem' OR 'self worth' OR lonely OR loneliness OR 'positive engagement' OR attitude OR benefit-finding OR meaning-making OR joy OR happy OR happiness OR excite* OR enthusiasm OR contentment OR psychosocial OR psycho-social OR self-efficacy OR smoking OR smoker OR cigarette* OR vaping OR vape OR e-cig OR ecig OR e-cigarette* OR ecigarette* OR tobacco OR marijuana OR cannabis OR THC OR nicotine OR opiate* OR heroin OR social OR companionship OR isolat* OR support* OR helpless* OR disappoint* OR symptom* OR sensorimotor OR friend* OR family OR families OR active OR activity OR sedentar* OR relationship* OR neuropsych* OR psycho* OR psychiatr* OR neurotic* OR neurocognit* OR QoL OR HRQoL OR nausea* OR vomit* OR emesis OR hyperemesis OR constipat* OR diarrhea OR thirst* OR itch OR itchy OR itching OR urination OR headache OR migraine* OR 'brain fog' OR pancreatitis OR (ulcer NEAR/3 (peptic OR stomach)) OR GERD OR gastroesophageal OR gastro-esophageal OR ((acid OR gastric) NEAR/3 reflux) OR osteopenia* OR osteoporos* OR polyuria OR nocturia OR nephrocalcinosis OR mephorlithaiasis OR CKD OR 'chronic kidney' OR hypertens* OR HTN OR 'high blood pressure' OR fibromyalgia OR pruritis OR neuralgi* OR ((muscle OR muscular OR musculoskeletal OR joint OR back OR neck OR stomach OR abdomen OR abdominal OR head) NEAR/3 (pain OR painful OR ache OR aching)) OR (quality NEAR/3 life)):ti,ab,kw<br>8. #5 OR #6 OR #7<br>9. #3 AND #4 AND #8 AND [embase]/lim AND ([article]/lim OR [article in press]/lim OR [data papers]/lim OR [review]/lim OR [preprint]/lim)
**WEB OF SCIENCE – SCI-EXP, SSCI, ESCI (CLARIVATE)**	1. (TI=(surgery OR surgical* OR parathyroidectom*)) OR (AB=(surgery OR surgical* OR parathyroidectom*)) OR (AK=(surgery OR surgical* OR parathyroidectom*)) OR<br>2. (TI=(denosumab OR biphosphonates OR cinacalcet)) OR (AB=(denosumab OR biphosphonates OR cinacalcet)) OR (AK=(denosumab OR biphosphonates OR cinacalcet))<br>3. #1 OR #2<br>4. (TI=((primary NEAR/1 (hyperparathyroid* OR hyper-parathyroid*)) OR pHPT)) OR (AB=((primary NEAR/1 (hyperparathyroid* OR hyper-

	parathyroid*)) OR pHPT)) OR (AK=((primary NEAR/1 (hyperparathyroid* OR hyper-parathyroid*)) OR pHPT))<br>5. (TI=(((cognitiv* OR cognition OR intelligence OR neurocognit* OR memory OR neuropsych* OR psycholog*) NEAR/3 (test* OR self-report* OR questionnaire* OR survey* OR answer* OR self-assess*)) OR psychometric* OR stanford-binnet OR wechsler OR PHQ OR ((patient OR patients) NEAR/3 (assess* OR report*) NEAR/3 (self OR outcome*)))) OR (AB=(((cognitiv* OR cognition OR intelligence OR neurocognit* OR memory OR neuropsych* OR psycholog*) NEAR/3 (test* OR self-report* OR questionnaire* OR survey* OR answer* OR self-assess*)) OR psychometric* OR stanford-binnet OR wechsler OR PHQ OR ((patient OR patients) NEAR/3 (assess* OR report*) NEAR/3 (self OR outcome*)))) OR (AK=(((cognitiv* OR cognition OR intelligence OR neurocognit* OR memory OR neuropsych* OR psycholog*) NEAR/3 (test* OR self-report* OR questionnaire* OR survey* OR answer* OR self-assess*)) OR psychometric* OR stanford-binnet OR wechsler OR PHQ OR ((patient OR patients) NEAR/3 (assess* OR report*) NEAR/3 (self OR outcome*))))<br>6. (TI=(((drug$ OR substance$) NEAR/3 (use$ OR abuse$ OR misuse$ OR problem* OR addict*)) OR (mental NEAR/3 (illness OR disorder*)) OR ((sex OR sexual) NEAR/3 (perform* OR interest* OR discomfort* OR function* OR impair* OR drive)) OR ((emotional* OR social* OR mental* OR physical*) NEAR/3 (health OR healthy OR wellness OR wellbeing OR well-being OR support OR safety OR isolat* OR engage*)) OR (social* NEAR/3 engage*) OR (daily NEAR/3 activit*) OR (medication NEAR/3 (complian* OR adheren*)) OR fatigue* OR exhaustion OR sleep* OR insomnia OR hypersomnia OR libido OR anxiety OR anxious OR depression OR depressed OR depressive OR somatoform OR alcohol* OR drinking OR anger OR angry OR cognition OR cognitive* OR "mental acuity" OR concentration OR memory OR "positive affect" OR "positive outlook" OR mood OR irritab* OR frustration OR "interpersonal sensitivity" OR envy OR disagreeable OR temper OR fear OR fearful* OR panic OR misery OR worry OR dread OR hyperarousal OR tension OR nervous* OR somatic OR dizzy OR dizziness OR sadness OR sad OR guilt* OR "self esteem" OR "self worth" OR lonely OR loneliness OR "positive engagement" OR attitude OR benefit-finding OR meaning-making OR joy OR happy OR happiness OR excite* OR enthusiasm OR contentment OR psychosocial OR psycho-social OR self-efficacy OR smoking OR smoker OR cigarette* OR vaping OR vape OR e-cig OR ecig OR e-cigarette* OR ecigarette* OR tobacco OR marijuana OR cannabis OR THC OR nicotine OR opiate* OR heroin OR social OR companionship OR isolat* OR support* OR ADL OR helpless* OR disappoint* OR symptom* OR sensorimotor OR friend* OR family OR families OR active OR activity OR sedentar* OR relationship* OR neuropsych* OR psycho* OR psychiatr* OR neurotic* OR neurocognit* OR QoL OR HRQoL OR nausea* OR vomit* OR emesis OR hyperemesis OR constipat* OR diarrhea OR thirst* OR itch OR itchy OR itching OR urination OR headache OR migraine* OR "brain fog" OR pancreatitis OR (ulcer NEAR/3 (peptic OR stomach)) OR GERD OR gastroesophageal OR gastro-esophageal OR ((acid OR gastric) NEAR/3 reflux) OR osteopenia* OR osteoporos* OR polyuria OR nocturia OR

	nephrocalcinosis OR mephorlithaiasis OR CKD OR "chronic kidney" OR hypertens* OR HTN OR "high blood pressure" OR fibromyalgia OR pruritis OR neuralgi* OR ((muscle OR muscular OR musculoskeletal OR joint OR back OR neck OR stomach OR abdomen OR abdominal OR head) NEAR/3 (pain OR painful OR ache OR aching)) OR (quality NEAR/3 life))) OR (AB=(((drug OR substance) NEAR/3 (use* OR abuse OR misuse OR problem OR addict*)) OR (mental NEAR/3 (illness OR disorder*)) OR ((sex OR sexual) NEAR/3 (perform* OR interest* OR discomfort* OR function* OR impair* OR drive)) OR ((emotional* OR social* OR mental* OR physical*) NEAR/3 (health OR healthy OR wellness OR wellbeing OR well-being OR support OR safety OR isolat* OR engage*)) OR (social* NEAR/3 engage*) OR (daily NEAR/3 activit*) OR (medication NEAR/3 (complian* OR adheren*)) OR fatigue* OR exhaustion OR sleep* OR insomnia OR hypersomnia OR libido OR anxiety OR anxious OR depression OR depressed OR depressive OR somatoform OR alcohol* OR drinking OR anger OR angry OR cognition OR cognitive* OR "mental acuity" OR concentration OR memory OR "positive affect" OR "positive outlook" OR mood OR irritab* OR frustration OR "interpersonal sensitivity" OR envy OR disagreeable OR temper OR fear OR fearful* OR panic OR misery OR worry OR dread OR hyperarousal OR tension OR nervous* OR somatic OR dizzy OR dizziness OR sadness OR sad OR guilt* OR "self esteem" OR "self worth" OR lonely OR loneliness OR "positive engagement" OR attitude OR benefit-finding OR meaning-making OR joy OR happy OR happiness OR excite* OR enthusiasm OR contentment OR psychosocial OR psycho-social OR self-efficacy OR smoking OR smoker OR cigarette* OR vaping OR vape OR e-cig OR ecig OR e-cigarette* OR ecigarette* OR tobacco OR marijuana OR cannabis OR THC OR nicotine OR opiate* OR heroin OR social OR companionship OR isolat* OR support* OR ADL OR helpless* OR disappoint* OR symptom* OR sensorimotor OR friend* OR family OR families OR active OR activity OR sedentar* OR relationship* OR neuropsych* OR psycho* OR psychiatr* OR neurotic* OR neurocognit* OR QoL OR HRQoL OR nausea* OR vomit* OR emesis OR hyperemesis OR constipat* OR diarrhea OR thirst* OR itch OR itchy OR itching OR urination OR headache OR migraine* OR "brain fog" OR pancreatitis OR (ulcer NEAR/3 (peptic OR stomach)) OR GERD OR gastroesophageal OR gastro-esophageal OR ((acid OR gastric) NEAR/3 reflux) OR osteopenia* OR osteoporos* OR polyuria OR nocturia OR nephrocalcinosis OR mephorlithaiasis OR CKD OR "chronic kidney" OR hypertens* OR HTN OR "high blood pressure" OR fibromyalgia OR pruritis OR neuralgi* OR ((muscle OR muscular OR musculoskeletal OR joint OR back OR neck OR stomach OR abdomen OR abdominal OR head) NEAR/3 (pain OR painful OR ache OR aching)) OR (quality NEAR/3 life))) OR (AK=(((drug OR substance) NEAR/3 (use* OR abuse OR misuse OR problem OR addict*)) OR (mental NEAR/3 (illness OR disorder*)) OR ((sex OR sexual) NEAR/3 (perform* OR interest* OR discomfort* OR function* OR impair* OR drive)) OR ((emotional* OR social* OR mental* OR physical*) NEAR/3 (health OR healthy OR wellness OR wellbeing OR well-being OR support OR safety OR isolat* OR engage*)) OR (social* NEAR/3 engage*) OR (daily NEAR/3 activit*) OR (medication NEAR/3 (complian* OR adheren*)) OR

	fatigue* OR exhaustion OR sleep* OR insomnia OR hypersomnia OR libido OR anxiety OR anxious OR depression OR depressed OR depressive OR somatoform OR alcohol* OR drinking OR anger OR angry OR cognition OR cognitive* OR "mental acuity" OR concentration OR memory OR "positive affect" OR "positive outlook" OR mood OR irritab* OR frustration OR "interpersonal sensitivity" OR envy OR disagreeable OR temper OR fear OR fearful* OR panic OR misery OR worry OR dread OR hyperarousal OR tension OR nervous* OR somatic OR dizzy OR dizziness OR sadness OR sad OR guilt* OR "self esteem" OR "self worth" OR lonely OR loneliness OR "positive engagement" OR attitude OR benefit-finding OR meaning-making OR joy OR happy OR happiness OR excite* OR enthusiasm OR contentment OR psychosocial OR psycho-social OR self-efficacy OR smoking OR smoker OR cigarette* OR vaping OR vape OR e-cig OR ecig OR e-cigarette* OR ecigarette* OR tobacco OR marijuana OR cannabis OR THC OR nicotine OR opiate* OR heroin OR social OR companionship OR isolat* OR support* OR ADL OR helpless* OR disappoint* OR symptom* OR sensorimotor OR friend* OR family OR families OR active OR activity OR sedentar* OR relationship* OR neuropsych* OR psycho* OR psychiatr* OR neurotic* OR neurocognit* OR QoL OR HRQoL OR nausea* OR vomit* OR emesis OR hyperemesis OR constipat* OR diarrhea OR thirst* OR itch OR itchy OR itching OR urination OR headache OR migraine* OR "brain fog" OR pancreatitis OR (ulcer NEAR/3 (peptic OR stomach)) OR GERD OR gastroesophageal OR gastro-esophageal OR ((acid OR gastric) NEAR/3 reflux) OR osteopenia* OR osteoporos* OR polyuria OR nocturia OR nephrocalcinosis OR mephorlithaiasis OR CKD OR "chronic kidney" OR hypertens* OR HTN OR "high blood pressure" OR fibromyalgia OR pruritis OR neuralgi* OR ((muscle OR muscular OR musculoskeletal OR joint OR back OR neck OR stomach OR abdomen OR abdominal OR head) NEAR/3 (pain OR painful OR ache OR aching)) OR (quality NEAR/3 life)))<br>7. #5 OR #6<br>8. #3 AND #4 AND #7
**CINAHL COMPLETE (EBSCO)**	S1 MH ("Parathyroid Glands/SU" OR "Surgery, Operative" OR "Surgery, Endocrine" OR "Hyperparathyroidism/SU") OR TI (parathyroidectom* OR surgery OR surgical*) OR AB (parathyroidectom* OR surgery OR surgical*)<br><br>S2 MH ("Parathyroid Glands/DT" OR "Hyperparathyroidism/DT") OR TI (denosumab OR biphosphonates OR cinacalcet) OR AB (denosumab OR biphosphonates OR cinacalcet)<br><br>S3 (S1 OR S2)<br><br>S4 MH "Hyperparathyroidism" OR TI ("Primary hyperparathyroidism" OR pHPT OR "Primary hyper-parathyroidism") OR AB ("Primary hyperparathyroidism" OR pHPT OR "Primary hyper-parathyroidism")<br><br>S5 MH ("Psychometrics OR "Neuropsychological Tests+" OR "Aptitude Tests+" OR TI (((cognitiv* OR cognition OR intelligence OR neurocognit*

OR memory OR neuropsych* OR psycholog*) N3 (test* OR self-report* OR questionnaire* OR survey* OR answer* OR self-assess*)) OR psychometric* OR stanford-binnet OR wechsler OR PHQ OR ((patient OR patients) N3 (assess* OR report*) N3 (self OR outcome*))) OR AB (((cognitiv* OR cognition OR intelligence OR neurocognit* OR memory OR neuropsych* OR psycholog*) N3 (test* OR self-report* OR questionnaire* OR survey* OR answer* OR self-assess*)) OR psychometric* OR stanford-binnet OR wechsler OR PHQ OR ((patient OR patients) N3 (assess* OR report*) N3 (self OR outcome*)))

S6 MH ("Mental Disorders+" OR "Mental Health" OR "Neuropsychology" OR "Social Behavior" OR "Social Integration" OR "Social Isolation+" OR "Social Participation" OR "Self Concept+" OR "Quality of Life+" OR "Psychological Well-Being" OR "Signs and Symptoms, Digestive+" OR "Fatigue+" OR "Mental Fatigue+" OR "Dysuria" OR "Pain" OR "Abdominal Pain+" OR "Arthralgia+" OR "Back Pain+" OR "Chronic Pain" OR "Headache" OR "Muscle Pain" OR "Neck Pain" OR "Neuralgia+" OR "Musculoskeletal Pain" OR "Polydipsia" OR "Sleepiness" OR "Hypertension+")

S7 TI (((drug OR substance) N3 (use* OR abuse OR misuse OR problem OR addict*)) OR (mental N3 (illness OR disorder*)) OR ((sex OR sexual) N3 (perform* OR interest* OR discomfort* OR function* OR impair* OR drive)) OR ((emotional* OR social* OR mental* OR physical*) N3 (health OR healthy OR wellness OR wellbeing OR well-being OR support OR safety OR isolat* OR engage*)) OR (social* N3 engage*) OR (daily N2 activit*) OR (medication N2 (complian* OR adheren*)) OR fatigue* OR exhaustion OR sleep* OR insomnia OR hypersomnia OR libido OR anxiety OR anxious OR depression OR depressed OR depressive OR somatoform OR alcohol* OR drinking OR anger OR angry OR cognition OR cognitive* OR "mental acuity" OR concentration OR memory OR "positive affect" OR "positive outlook" OR mood OR irritab* OR frustration OR "interpersonal sensitivity" OR envy OR disagreeable OR temper OR fear OR fearful* OR panic OR misery OR worry OR dread OR hyperarousal OR tension OR nervous* OR somatic OR dizzy OR dizziness OR sadness OR sad OR guilt* OR "self esteem" OR "self worth" OR lonely OR loneliness OR "positive engagement" OR attitude OR benefit-finding OR meaning-making OR joy OR happy OR happiness OR excite* OR enthusiasm OR contentment OR psychosocial OR psycho-social OR self-efficacy OR smoking OR smoker OR cigarette* OR vaping OR vape OR e-cig OR ecig OR e-cigarette* OR ecigarette* OR tobacco OR marijuana OR cannabis OR THC OR nicotine OR opiate* OR heroin OR social OR companionship OR isolat* OR support* OR ADL OR helpless* OR disappoint* OR symptom* OR sensorimotor OR friend* OR family OR families OR active OR activity OR sedentar* OR relationship* OR neuropsych* OR psycho* OR psychiatr* OR neurotic* OR neurocognit* OR QoL OR HRQoL OR nausea* OR vomit* OR emesis OR hyperemesis OR constipat* OR diarrhea OR thirst* OR itch OR itchy OR itching OR urination OR headache OR migraine* OR "brain

fog" OR pancreatitis OR (ulcer N3 (peptic OR stomach)) OR GERD OR gastroesophageal OR gastro-esophageal OR ((acid OR gastric) N3 reflux) OR osteopenia* OR osteoporos* OR polyuria OR nocturia OR nephrocalcinosis OR mephorlithaiasis OR CKD OR "chronic kidney" OR hypertens* OR HTN OR "high blood pressure" OR fibromyalgia OR pruritis OR neuralgi* OR ((muscle OR muscular OR musculoskeletal OR joint OR back OR neck OR stomach OR abdomen OR abdominal OR head) N3 (pain OR painful OR ache OR aching)) OR (quality N3 life))
OR
AB (((drug OR substance) N3 (use* OR abuse OR misuse OR problem OR addict*)) OR (mental N3 (illness OR disorder*)) OR ((sex OR sexual) N3 (perform* OR interest* OR discomfort* OR function* OR impair* OR drive)) OR ((emotional* OR social* OR mental* OR physical*) N3 (health OR healthy OR wellness OR wellbeing OR well-being OR support OR safety OR isolat* OR engage*)) OR (social* N3 engage*) OR (daily N2 activit*) OR (medication N2 (complian* OR adheren*)) OR fatigue* OR exhaustion OR sleep* OR insomnia OR hypersomnia OR libido OR anxiety OR anxious OR depression OR depressed OR depressive OR somatoform OR alcohol* OR drinking OR anger OR angry OR cognition OR cognitive* OR "mental acuity" OR concentration OR memory OR "positive affect" OR "positive outlook" OR mood OR irritab* OR frustration OR "interpersonal sensitivity" OR envy OR disagreeable OR temper OR fear OR fearful* OR panic OR misery OR worry OR dread OR hyperarousal OR tension OR nervous* OR somatic OR dizzy OR dizziness OR sadness OR sad OR guilt* OR "self esteem" OR "self worth" OR lonely OR loneliness OR "positive engagement" OR attitude OR benefit-finding OR meaning-making OR joy OR happy OR happiness OR excite* OR enthusiasm OR contentment OR psychosocial OR psycho-social OR self-efficacy OR smoking OR smoker OR cigarette* OR vaping OR vape OR e-cig OR ecig OR e-cigarette* OR ecigarette* OR tobacco OR marijuana OR cannabis OR THC OR nicotine OR opiate* OR heroin OR social OR companionship OR isolat* OR support* OR ADL OR helpless* OR disappoint* OR symptom* OR sensorimotor OR friend* OR family OR families OR active OR activity OR sedentar* OR relationship* OR neuropsych* OR psycho* OR psychiatr* OR neurotic* OR neurocognit* OR QoL OR HRQoL OR nausea* OR vomit* OR emesis OR hyperemesis OR constipat* OR diarrhea OR thirst* OR itch OR itchy OR itching OR urination OR headache OR migraine* OR "brain fog" OR pancreatitis OR (ulcer N3 (peptic OR stomach)) OR GERD OR gastroesophageal OR gastro-esophageal OR ((acid OR gastric) N3 reflux) OR osteopenia* OR osteoporos* OR polyuria OR nocturia OR nephrocalcinosis OR mephorlithaiasis OR CKD OR "chronic kidney" OR hypertens* OR HTN OR "high blood pressure" OR fibromyalgia OR pruritis OR neuralgi* OR ((muscle OR muscular OR musculoskeletal OR joint OR back OR neck OR stomach OR abdomen OR abdominal OR head) N3 (pain OR painful OR ache OR aching)) OR (quality N3 life))

S8 (S5 OR S6 OR S7)

S9 (S3 AND S4 AND S8)

**COCHRANE LIBRARY (WILEY)**	(hyperparathyroid* OR (hyper NEXT parathyroid*) OR pHPT OR HPT):ti,ab,kw AND ((parathyroid* NEAR/3 (surgery OR surgical)) OR parathyroidectomy OR calcimimetic* OR cincalcet OR sensipar):ti,ab,kw
**APA PSYCINFO (EBSCO)**	DE (("Parathyroid Disorders" OR "Parathyroid Hormone") AND ("Surgery" OR "Drug Therapy")) OR TI(hyperparathyroid* OR hyper-parathyroid* OR pHPT OR ((surgery OR surgical* OR cinacalcet OR denosumab OR biphosphonat*) N3 parathyroid*) OR parathyroidectom*) OR AB(hyperparathyroid* OR hyper-parathyroid* OR pHPT OR ((surgery OR surgical* OR cinacalcet OR denosumab OR biphosphonat*) N3 parathyroid*) OR parathyroidectom*)
