# Supplementary material for: Patient-Reported Outcome Measures Used in Primary Hyperparathyroidism: A Scoping Review
Source: Ann Surg Oncol. 2026 Feb 12;33(4):3435–48. doi: 10.1245/s10434-026-19246-4 (PMC12982322; doi:10.1245/s10434-026-19246-4)
Supplement: Supplementary file 2 — Supplementary file2 (PDF 302 KB) [file 10434_2026_19246_MOESM2_ESM.pdf]

First Author	Year	Country	Title	DOI
Aberg	2015	Sweden	Health-related quality of life after successful surgery for primary hyperparathyroidism: no additive effect from vitamin D supplementation: results of a double-blind randomized study.	10.1530/EJE-14-0757
Adler	2008	USA	The influence of surgical approach on quality of life after parathyroid surgery.	10.1245/s10434-008-9879-0
Adler	2009	USA	Surgery improves quality of life in patients with "mild" hyperparathyroidism	10.1016/j.amjsurg.2008.09.009
Ambrogini	2007	Italy	Surgery or surveillance for mild asymptomatic primary hyperparathyroidism: a prospective, randomized clinical trial.	10.1210/jc.2007-0219
Amstrup	2011	Denmark	Patients with surgically cured primary hyperparathyroidism have a reduced quality of life compared with population-based healthy sex-, age-, and season-matched controls.	10.1530/EJE-11-0301
Aspinall	2010	Australia, UK	Long-term symptom relief from primary hyperparathyroidism following minimally invasive parathyroidectomy.	10.1007/s00268-010-0687-x
Babińska	2011	Poland	Evaluation of selected cognitive functions before and after surgery for primary hyperparathyroidism.	10.1007/s00423-011-0885-5
Bannani	2018	France	Effect of parathyroidectomy on quality of life and non-specific symptoms in normocalcaemic primary hyperparathyroidism.	10.1002/bjs.10739
Barczynski	2006	Poland	Minimally invasive video-assisted parathyroidectomy versus open minimally invasive parathyroidectomy for a solitary parathyroid adenoma: a prospective, randomized, blinded trial.	10.1007/s00268-005-0312-6
Baugh	2024	USA	Sex differences in patients with primary hyperparathyroidism.	10.1016/j.surg.2023.07.044
Benge	2009	USA	Cognitive and affective sequelae of primary hyperparathyroidism and early response to parathyroidectomy.	10.1017/S1355617709990695
Blanchard	2014	France	Quality of life is modestly improved in older patients with mild primary hyperparathyroidism postoperatively: results of a prospective multicenter study.	10.1245/s10434-014-3731-5
Bollerslev	2007	Denmark, Norway, Sweden	Medical observation, compared with parathyroidectomy, for asymptomatic primary hyperparathyroidism: a prospective, randomized trial.	10.1210/jc.2006-1836
Brescia	2022	Brazil, Netherlands	Impact of parathyroidectomy on quality of life in multiple endocrine neoplasia type 1.	10.1530/EC-22-0021
Burney	1996	USA	Assessment of patient outcomes after operation for primary hyperparathyroidism.	10.1016/s0039-6060(96)80048-1
Burney	1998	USA	Surgical correction of primary hyperparathyroidism improves quality of life.	10.1016/S0039-6060(98)70039-X
Caillard	2007	France	Prospective evaluation of quality of life (SF-36v2) and nonspecific symptoms before and after cure of primary hyperparathyroidism (1-year follow-up).	10.1016/j.surg.2006.12.004
Caton	2018	UK	Quality of life improvement following parathyroid surgery: A preliminary 3-year review of 56 patients from a single surgical centre.	10.1111/coa.13276
Chan	2024	USA	Actigraphy measures show sleep improvement after parathyroidectomy for primary hyperparathyroidism.	10.1016/j.amjoto.2024.104297
Chiang	2004	Australia	A controlled, prospective study of neuropsychological outcomes post parathyroidectomy in primary hyperparathyroid patients.	10.1111/j.1365-2265.2004.02180.x
Christensen	2022	Denmark	Changes in quality of life 6 months after parathyroidectomy for primary hyperparathyroidism.	10.1530/EC-21-0630
Dulfer	2016	The Netherlands	Impact of parathyroidectomy for primary hyperparathyroidism on quality of life: A case-control study using Short Form Health Survey 36.	10.1002/hed.24499
Edwards	2006	USA	Improvement in the health-related quality-of-life symptoms of hyperparathyroidism is durable on long-term follow-up.	10.1016/j.surg.2006.06.016
Egan	2006	USA	Parathyroidectomy for primary hyperparathyroidism in octogenarians and nonagenarians: a risk-benefit analysis.	10.1016/j.jss.2007.01.027
Ejlsmark-Svensson	2018	Denmark	Health-related quality of life improves 1 year after parathyroidectomy in primary hyperparathyroidism: A prospective cohort study.	10.1111/cen.13865
Espiritu	2011	USA	Depression in primary hyperparathyroidism: prevalence and benefit of surgery.	10.1210/jc.2011-1486
Febrero	2023	Spain	The Influence of Hyperparathyroidism Patient Profile on Quality of Life After Parathyroidectomy.	10.1007/s00268-023-07066-6

Febrero	2024	Spain	Improvement of mood and sleep quality in patients with primary hyperparathyroidism after parathyroidectomy: A prospective case-control study.	10.1016/j.surg.2024.01.003
Frey	2023	France	Parathyroidectomy for primary hyperparathyroidism: effect on quality of life after 3 years - a prospective cohort study.	10.1097/JS9.0000000000000282
Gladkova	2023	Russia	Quality of life in patients with primary hyperparathyroidism after parathyroidectomy: long term single center experience	10.1186/s12902-023-01344-z
Gopinath	2010	UK	Persistent symptomatic improvement in the majority of patients undergoing parathyroidectomy for primary hyperparathyroidism.	10.1007/s00423-010-0689-z
Greutelaers	2004	Australia, Canada	Pasieka Illness Questionnaire: its value in primary hyperparathyroidism.	10.1111/j.1445-2197.2004.02907.x
Hasse	2000	Germany	How asymptomatic is asymptomatic primary hyperparathyroidism?	10.1055/s-2000-7754
Hasse	2002	Germany	Quality of life and patient satisfaction after reoperation for primary hyperparathyroidism: analysis of long-term results.	10.1007/s00268-002-6664-2
Hermsen	2014	Germany	Perioperative changes in cortical excitability, mood, and quality of life in patients with primary hyperparathyroidism: a pilot study using transcranial magnetic stimulation.	10.1530/EJE-13-0552
Jones	2000	USA	Patient expectations for surgery: are they being met?	10.1016/s1070-3241(00)26029-x
Jovanovic	2023	Serbia	Effects of successful parathyroidectomy on neuropsychological and cognitive status in patients with asymptomatic primary hyperparathyroidism.	10.1007/s12020-023-03426-3
Kahal	2012	UK	The effect of parathyroidectomy on neuropsychological symptoms and biochemical parameters in patients with asymptomatic primary hyperparathyroidism.	10.1111/j.1365-2265.2011.04197.x
Kearns	2019	USA	Clinical characteristics and depression score response after parathyroidectomy in primary hyperparathyroidism.	10.1111/cen.14045
Koman	2019	Sweden	Short-term medical treatment of hypercalcaemia in primary hyperparathyroidism predicts symptomatic response after parathyroidectomy.	10.1093/bjsopen/zraa029
Koman	2020	Sweden	Prediction of cognitive response to surgery in elderly patients with primary hyperparathyroidism.	10.1002/bjs.11319
La	2017	USA	Parathyroidectomy for primary hyperparathyroidism improves sleep quality: A prospective study	10.1016/j.surg.2016.05.047
Leong	2009	UK	Health-related quality of life improvement following surgical treatment of primary hyperparathyroidism in a United Kingdom population.	10.1016/j.surge.2009.10.005
Liu	2020	USA	Cognition and cerebrovascular function in primary hyperparathyroidism before and after parathyroidectomy.	10.1016/j.surg.2020.06.006
Liu	2021	USA	Neuropsychologic changes in primary hyperparathyroidism after parathyroidectomy from a dual-institution prospective study.	10.1007/s40618-019-01128-0
Ljunghall	1991	Sweden	Longitudinal studies of mild primary hyperparathyroidism.	10.1002/jbmr.5650061423
Mihai	2008	UK	Pasieka's parathyroid symptoms scores correlate with SF-36 scores in patients undergoing surgery for primary hyperparathyroidism.	10.1007/s00268-008-9509-9
Mittendorf	2007	USA	Improvement of sleep disturbance and neurocognitive function after parathyroidectomy in patients with primary hyperparathyroidism.	10.4158/EP.13.4.338
Mohan	2021	India	Quality of life assessment after parathyroidectomy in symptomatic primary hyperparathyroidism using the SF-36 questionnaire.	10.47717/turkjsurg.2021.5156
Murray	2014	USA	Improvement of sleep disturbance and insomnia following parathyroidectomy for primary hyperparathyroidism.	10.1007/s00268-013-2285-1
Norman	2015	USA	Surgical cure of primary hyperparathyroidism ameliorates gastroesophageal reflux symptoms.	10.1007/s00268-014-2876-5
Okamoto	2002	Japan	Outcome study of psychological distress and nonspecific symptoms in patients with mild primary hyperparathyroidism.	10.1001/archsurg.137.7.779

Papavramidis	2022	Greece	The impact of age on quality of life and frailty outcomes after parathyroidectomy in patients with primary hyperparathyroidism.	10.1007/s40618-021-01710-5
Pasieka	1998	Canada	Prospective surgical outcome study of relief of symptoms following surgery in patients with primary hyperparathyroidism.	10.1007/s002689900428
Pasieka	2002	Australia, Canada, USA	Patient-based surgical outcome tool demonstrating alleviation of symptoms following parathyroidectomy in patients with primary hyperparathyroidism.	10.1007/s00268-002-6623-y
Pasieka	2009	Canada	The long-term benefit of parathyroidectomy in primary hyperparathyroidism: a 10-year prospective surgical outcome study.	10.1016/j.surg.2009.10.021
Perrier	2006	USA	Preliminary report: functional MRI of the brain may be the ideal tool for evaluating neuropsychologic and sleep complaints of patients with primary hyperparathyroidism.	10.1007/s00268-005-0361-x
Perrier	2009	USA	Prospective, randomized, controlled trial of parathyroidectomy versus observation in patients with "asymptomatic" primary hyperparathyroidism.	10.1016/j.surg.2009.09.034
Pinchot	2012	USA	Changes in swallowing-related quality of life after parathyroidectomy for hyperparathyroidism: a prospective cohort study.	10.1634/theoncologist.2012-0203
Pretorius	2021	Denmark, Norway, Sweden	Effects of Parathyroidectomy on Quality of Life: 10 Years of Data From a Prospective Randomized Controlled Trial on Primary Hyperparathyroidism (the SIPH-Study).	10.1002/jbmr.4199
Quiros	2003	USA	Health-related quality of life in hyperparathyroidism measurably improves after parathyroidectomy.	10.1016/s0039-6060(03)00316-7
Ramakant	2011	India	Salutary effect of parathyroidectomy on neuropsychiatric symptoms in patients with primary hyperparathyroidism: evaluation using PAS and SF-36v2 scoring systems.	10.4103/0022-3859.81859
Rao	2004	USA	Randomized controlled clinical trial of surgery versus no surgery in patients with mild asymptomatic primary hyperparathyroidism.	10.1210/jc.2004-0028
Reiher	2012	USA	Symptoms of gastroesophageal reflux disease improve after parathyroidectomy.	10.1016/j.surg.2012.08.051
Rolighed	2014	Denmark	Muscle function is impaired in patients with "asymptomatic" primary hyperparathyroidism.	10.1007/s00268-013-2273-5
Rolighed	2015	Denmark	No beneficial effects of vitamin D supplementation on muscle function or quality of life in primary hyperparathyroidism: results from a randomized controlled trial.	10.1530/EJE-14-0940
Roman	2005	USA	Parathyroidectomy improves neurocognitive deficits in patients with primary hyperparathyroidism.	10.1016/j.surg.2005.08.033
Roman	2011	USA	The effects of serum calcium and parathyroid hormone changes on psychological and cognitive function in patients undergoing parathyroidectomy for primary hyperparathyroidism.	10.1097/SLA.0b013e3181f66720
Ryhanen	2015	Finland	Health-related quality of life is impaired in primary hyperparathyroidism and significantly improves after surgery: a prospective study using the 15D instrument.	10.1530/EC-15-0053
Sevinc	2022	Turkey	Evaluation of Swallowing Related Quality of Life and Health Related Quality of Life in Patients with Primary Hyperparathyroidism	10.1007/s12262-022-03549-3
Shah-Becker	2018	USA	Early neurocognitive improvements following parathyroidectomy for primary hyperparathyroidism.	10.1002/lary.26617
Sheldon	2002	USA	Surgical treatment of hyperparathyroidism improves health-related quality of life.	10.1001/archsurg.137.9.1022
Slepavicius	2008	Lithuania	Focused versus conventional parathyroidectomy for primary hyperparathyroidism: a prospective, randomized, blinded trial.	10.1007/s00423-008-0408-1
Slitt	2005	USA	Hyperparathyroidism but a negative sestamibi scan: a clinical dilemma.	10.1016/j.amjsurg.2005.07.007
Solomon	1994	USA	Psychologic symptoms before and after parathyroid surgery.	10.1016/0002-9343(94)90128-7
Somuncu	2021	Turkey	The effect of parathyroidectomy on quality of life in primary hyperparathyroidism: evaluation with using sf-36 and phpqol questionnaire.	10.1507/endocrj.EJ20-0417
Stechman	2009	UK	Parathyroidectomy is safe and improves symptoms in elderly patients with primary hyperparathyroidism (PHPT).	10.1111/j.1365-2265.2009.03540.x

Storvall	2017	Finland	Surgery Significantly Improves Neurocognition, Sleep, and Blood Pressure in Primary Hyperparathyroidism: A 3-Year Prospective Follow-Up Study.	10.1055/s-0043-118347
Sywak	2002	Canada	Do the National Institutes of Health consensus guidelines for parathyroidectomy predict symptom severity and surgical outcome in patients with primary hyperparathyroidism?.	10.1067/msy.2002.128693
Szalat	2022	Israel	Successful parathyroidectomy improves cognition in patients with primary hyperparathyroidism: A prospective study in a tertiary medical center and comprehensive review of the literature.	10.3389/fendo.2022.1095189
Talpos	2000	USA	Randomized trial of parathyroidectomy in mild asymptomatic primary hyperparathyroidism: patient description and effects on the SF-36 health survey.	10.1067/msy.2000.110844
Tang	2007	Australia	Does the surgical approach affect quality of life outcomes?--a comparison of minimally invasive parathyroidectomy with open parathyroidectomy.	10.1016/j.ijsu.2006.01.008
Tolley	2011	UK	Robotic-assisted parathyroidectomy: a feasibility study.	10.1177/0194599811402152
Tolley	2015	UK	Long-term prospective evaluation comparing robotic parathyroidectomy with minimally invasive open parathyroidectomy for primary hyperparathyroidism.	10.1002/hed.23990
Trombetti	2016	Switzerland	Clinical presentation and management of patients with primary hyperparathyroidism of the Swiss Primary Hyperparathyroidism Cohort: a focus on neuro-behavioral and cognitive symptoms.	10.1007/s40618-015-0423-3
Tzikos	2021	Greece	Quality of Life in Patients With Asymptomatic Primary Hyperparathyroidism After Parathyroidectomy: A 3-Year Longitudinal Study.	10.1016/j.eprac.2021.01.003
Vadhwana	2021	UK	Impact on Quality of Life After Parathyroidectomy for Asymptomatic Primary Hyperparathyroidism.	10.1016/j.jss.2020.12.023
Venkat	2012	USA	Long-term outcome in patients with primary hyperparathyroidism who underwent minimally invasive parathyroidectomy	10.1007/s00268-011-1344-8
Vera	2014	Italy	Five-year longitudinal evaluation of mild primary hyperparathyroidism - medical treatment versus clinical observation.	10.5603/EP.2014.0063
Walker	2004	USA	Symptoms in patients with primary hyperparathyroidism: muscle weakness or sleepiness.	10.1210/jc.2008-2574
Walker	2009	USA	Neuropsychological features in primary hyperparathyroidism: a prospective study.	10.4158/EP.10.5.404
Wang	2023	China	PTH levels, sleep quality, and cognitive function in primary hyperparathyroidism.	10.1007/s12020-023-03410-x
Weber	2007	Germany	Effect of parathyroidectomy on quality of life and neuropsychological symptoms in primary hyperparathyroidism.	10.1007/s00268-007-9006-6
Weber	2013	Germany	Parathyroidectomy, Elevated Depression Scores, and Suicidal Ideation in Patients With Primary Hyperparathyroidism Results of a Prospective Multicenter Study	10.1001/2013.jamasurg.316
Zanocco	2015	USA	Improvement in patient-reported physical and mental health after parathyroidectomy for primary hyperparathyroidism.	10.1016/j.surg.2015.03.054
Zheng	2024	USA	Improved sexual function after parathyroidectomy in women with primary hyperparathyroidism.	10.1016/j.amjsurg.2023.11.002
